# Clinical effects of transforaminal approach vs interlaminar approach in treating lumbar disc herniation: A clinical study protocol

**DOI:** 10.1097/MD.0000000000022701

**Published:** 2020-10-30

**Authors:** Wei Chen, Yong Zheng, Guiqing Liang, Guangfu Chen, Yabin Hu

**Affiliations:** a Department of Spine Surgery, Fuzhou Second Hospital Affiliated to Xiamen University, Fujian; b Department of Bone Injury, Shaanxi Hospital of Traditional Chinese Medicine, Shaanxi; c Department of Anesthesiology, Fuzhou Second Hospital Affiliated to Xiamen University, Fujian; d Department of Orthopedics, The Second Hospital of Nanjing, Jiangsu, China.

**Keywords:** interlaminar approach, lumbar disc herniation, percutaneous endoscopic lumbar discectomy, protocol, transforaminal approach

## Abstract

**Background:**

Percutaneous endoscopic lumbar discectomy (PELD) has routinely performed in recent years for lumbar disc herniation because of the advances in technology of minimally invasive spine surgery. Two common operating routes for PELD have been introduced in the literature: transforaminal approach (TA) and interlaminar approach (IA). The purpose of our current retrospective clinical trial was to study whether the effect of IA-PELD is better than TA-PELD in the incidence of complications and clinical prognosis scores in the patients with L5-S1 lumbar disc herniation.

**Methods:**

Our present research was approved by the institutional review board in the Second Hospital of Nanjing. All the patients offered the informed consent. All the procedures containing human participants were conducted on the basis of the Helsinki Declaration. A retrospective analysis was implemented on 126 patients with L5-S1 disc herniated radiculopathy from March 2016 to March 2018, who were treated with the PELD utilizing the IA technique or the TA technique. Relevant data, such as the patients demographics, surgical duration, length of hospital stay, hospitalization expenses, complications were recorded. In our work, the outcomes of patients were determined at baseline, 6 months, 12 months, and 24 months after treatment. The measure of primary outcome was Oswestry Disability Index score. The other outcomes measured were Numeric Rating Scale pain scale, surgical duration, length of hospital stay, and complications. The software of SPSS Version 22.0 (IBM Corporation, Armonk, NY) was applied for the analysis of all the statistical data. When *P* value <.05, it was considered to be significant in statistics.

**Results:**

This protocol will provide a solid theoretical basis for exploring which PELD approach is better in treatment of lumbar disc herniation.

**Trial registration:**

This study protocol was registered in Research Registry (researchregistry5988).

## 1 Introduction

The pain in the leg and back after lumbar disc herniation is the result of compression and an uncomfortable process caused by a herniated disc.^[1–3]^ It is a kind of familiar disease, and its prevalence rate is approximately 2% to 5% in general population. If the conservative treatment fails, or if the disease has a significant impact on the daily activities, surgery becomes a significant way for these patients.^[4]^ Percutaneous endoscopic lumbar discectomy (PELD) has routinely performed in recent years for lumbar disc herniation because of the advances in technology of minimally invasive spine surgery. PELD has the advantages of shorter operation time, less bleeding, and less incision, shorter bed rest time, faster functional recovery, and less injury of paravertebral muscle, as well as satisfactory functional results.^[5–10]^

Two common operating routes for PELD have been introduced in the literature: transforaminal approach (TA) and interlaminar approach (IA). TA is a kind of minimally invasive spine surgery, it remains the spine stable and avoids the formation of epidural scar.^[11–13]^ In view of the anatomic features of the L5-S1, namely wide interlaminar space, narrow intervertebral foramen and pelvic wing, many surgeons question the efficacy of TA on L5-S1.^[14–17]^ Nevertheless, some scholars believe that the TA-PELD can reach all the lumbar levels, even L5-S1. On the other hand, the wide L5-S1 interlaminar space offers excellent working space for IA, and the former researches have achieved good clinical outcomes with the IA-PELD at L5-S1 level.^[18–20]^

Despite some researchers have compared the safety and effectiveness of TA-PELD and IA-PELD for the lumbar disc herniation of L5-S1 levels; however, they got conflicting outcomes in several variables.^[10,14–19]^ Thus, the purpose of our current retrospective clinical trial was to study whether the effect of IA-PELD is better than TA-PELD in the incidence of complications and clinical prognosis scores in the patients with L5-S1 lumbar disc herniation.

## 2 Materials and methods

### 2.1 Screening criteria

The inclusion criteria for our investigation included:

(1) symptomatic radiation pain of leg was more common than the back pain, and the straight leg elevation test was positive;
(2) magnetic resonance imaging and computed tomography showed that the single level disc herniation at the level of L5-S1 was associated with clinical manifestations;
(3) there was no significant remission after 6 weeks of conventional expected treatment; and
(4) no former history of lumbar surgery at same level.

The exclusion criteria were as follows:

(1) intraspinal stenosis,
(2) recurrence of disc herniation occurred at same level
(3) segmental instability, and
(4) the coexisting pathological, state, for example, fracture, tumor, or infection.

### 2.2 Study design

Our present research was approved by the institutional review board in the Second Hospital of Nanjing (JS2020011204). All the patients offered the informed consent. All the procedures containing human participants were conducted on the basis of the Helsinki Declaration. A retrospective analysis was implemented on 126 patients with L5-S1 disc herniated radiculopathy from March 2016 to March 2018, who were treated with the PELD utilizing the IA technique or the TA technique. Our study protocol was registered in research registry (researchregistry5988).

### 2.3 Surgical procedures

#### 2.3.1 TA technique

TA-PELD was conducted under the condition of local anesthesia and the patient was prone to the radiolucent table. The operative segment and insertion point of the puncture needle were determined under the fluoroscopy of anterior-posterior and lateral C-arm. The puncture needle was inserted into the intervertebral disc at 15°–25° angle with the horizontal plane at a distance of 12 to 15 cm from midline. As the needle is inserted into the center of the disc, the core of the needle is removed and the mixture of Methylthioninium Chloride and contrast agent was injected into disc. The guide wire passes through the 18-gauge needle and makes a 7 mm incision in skin where it enters. The cannula and dilator were then inserted. Under the endoscope, the herniated disc was removed with the rongeurs and endoscopic forceps until the traversing nerve root relieved completely.

#### 2.3.2 IA technique

IA-PELD was performed under general anesthesia. Under the guidance of anterior-posterior fluoroscopy, the posterior midline of L5-S1 segment was clearly marked. After a small incision in the fascia and skin, a dilator was inserted and anchored at the L5 lamina lower edge. The working channel was introduced on the expander and the ultimate position was checked on the lateral perspective images and AP. Soft tissue was cleared, containing paravertebral muscles, and the ligamentum flavum was exposed, and a hole was made in the ligamentum flavum. The working cannula is inserted through this hole into epidural space to expose the nerve roots and epidural edges. In the case of slight root canal retraction, epidural dissection was conducted. The sequestrated and protruded disc pieces were found and then removed through utilizing the disc forceps. Root mobility was examined after the pathological disc was removed.

### 2.4 Outcomes

Relevant data, such as the patients demographics, surgical duration, length of hospital stay, hospitalization expenses, complications were recorded. In our work, the outcomes of patients were determined at baseline, 6 months, 12 months, and 24 months after treatment. The measure of primary outcome was Oswestry Disability Index (ODI) score. The other outcomes measured were Numeric Rating Scale (NRS) pain scale, surgical duration, length of hospital stay, and complications. ODI includes 10 items about the severity of leg or back diseases that influence the ability to manage daily living. These 10 components include the daily functions and pain (containing personal hygiene, pain intensity, sitting, walking, lifting, sleeping, standing, and traveling, sexual activity, as well as social activity). Each item will be scored on a six-point scale (0–5); the higher the score, the higher the degree of disability associated with the lower back pain (Table 1).

**Table 1:**
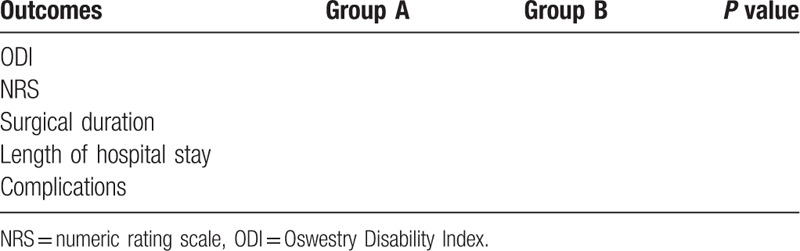
Postoperative outcomes.

### 2.5 Statistical analysis

The software of SPSS Version 22.0 (IBM Corporation, Armonk, NY) was applied for the analysis of all the statistical data. The mean ± SD was utilized to express the quantitative data. The t test and sample means were used for the comparison between groups. The χ^2^ test was applied for the comparison between the complications, axillar disc size and sex composition. And the comparison of ODI and NRS before and after the operation between the groups was carried out with the paired t test. The Mann-Whitney U test was utilized to compare the macnab criteria. When *P* value < .05, it was considered to be significant in statistics.

## 3 Discussion

The purpose of our current retrospective clinical trial was to study whether the effect of IA-PELD is better than TA-PELD in the incidence of complications and clinical prognosis scores in the patients with L5-S1 lumbar disc herniation. Our present investigation requires the collection and the analyses of retrospective data, which may allow the patients choose confusion and bias. The secondary limitation is that the number of patients assessing the multiple variables is relatively small. Third, the follow-up time is short, and the long-term efficacy needs to be in-depth observed.

## Author contributions

**Conceptualization:** Wei Chen, Yong Zheng, Guiqing Liang.

**Data curation:** Wei Chen, Yong Zheng.

**Formal analysis:** Wei Chen, Yong Zheng, Guiqing Liang, Guangfu Chen.

**Funding acquisition:** Yabin Hu.

**Investigation:** Wei Chen.

**Methodology:** Guangfu Chen.

**Project administration:** Guangfu Chen.

**Resources:** Yabin Hu.

**Software:** Guiqing Liang.

**Supervision:** Guiqing Liang, Yabin Hu.

**Validation:** Yong Zheng.

**Visualization:** Guiqing Liang.

**Writing – original draft:** Wei Chen.

**Writing – review & editing:** Yabin Hu.
